# Supplementary material for: Evaluation of microbiome enrichment and host DNA depletion in human vaginal samples using Oxford Nanopore’s adaptive sequencing
Source: Sci Rep. 2022 Mar 7;12:4000. doi: 10.1038/s41598-022-08003-8 (PMC8901746; doi:10.1038/s41598-022-08003-8)
Supplement: Supplementary file 1 — Supplementary Figures. [file 41598_2022_8003_MOESM1_ESM.docx]

Supplementary Material

# Supplementary Figures


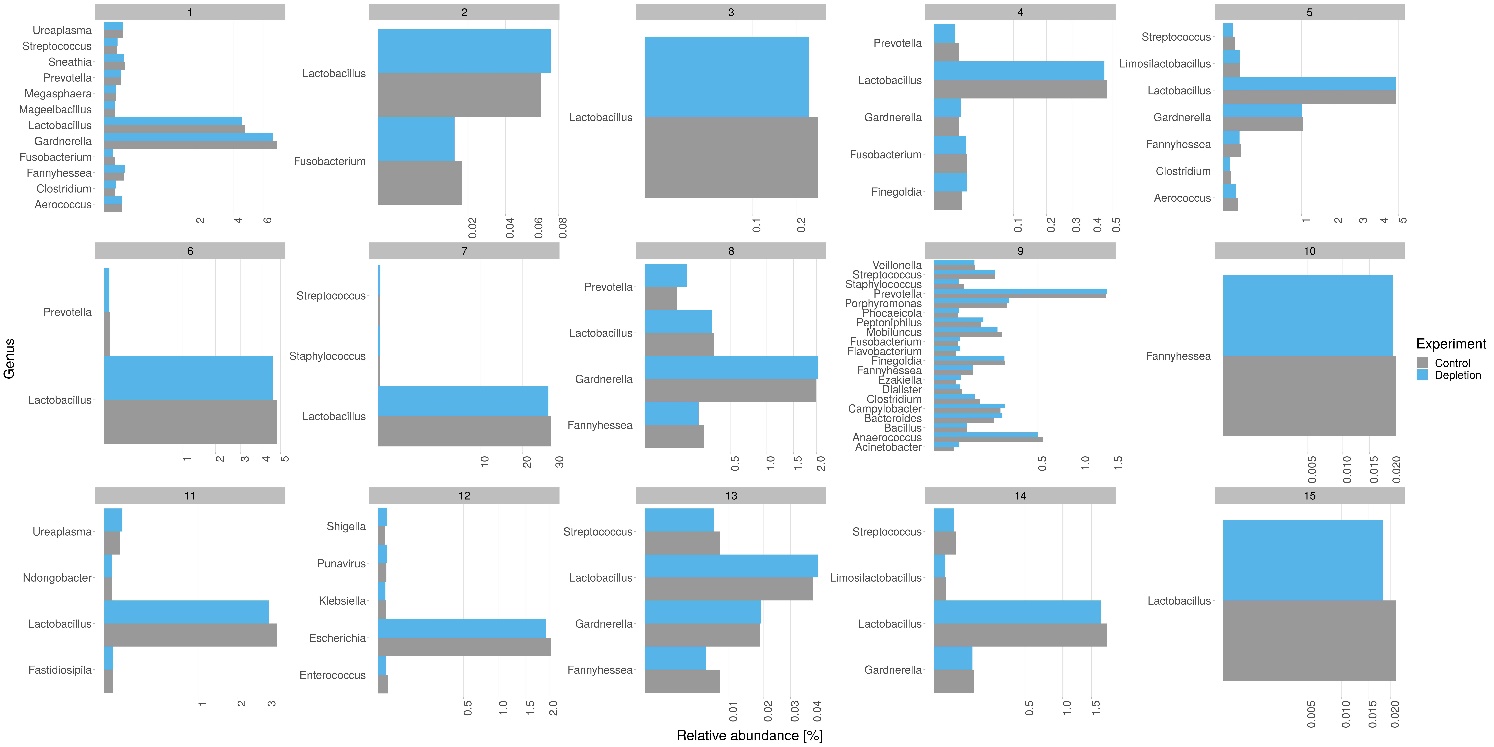


*Figure S1: Comparison of the relative abundance of bacterial genera for all vaginal samples (control in grey, depletion in blue).*

*
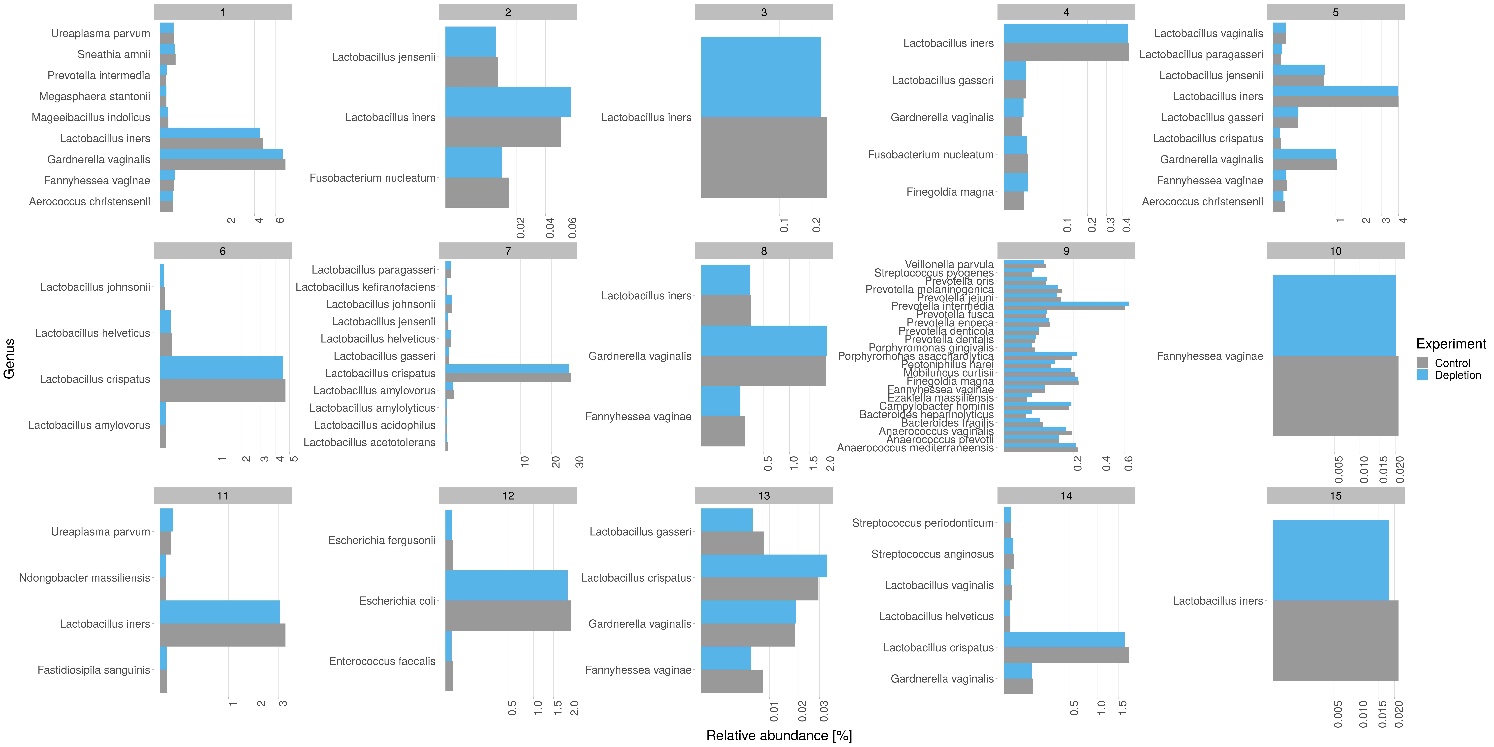
*

*Figure S2: Comparison of the relative abundance of bacterial species for all vaginal samples (control in grey, depletion in blue).*
